# N_3C_-Defect-Tuned g-C_3_N_4_ Photocatalysts: Structural Optimization and Enhanced Tetracycline Degradation Performance

**DOI:** 10.3390/nano15060466

**Published:** 2025-03-19

**Authors:** Yu Lu, Chengbao Liu, Leizhi Zheng, Feng Chen, Junchao Qian, Xianrong Meng, Zhigang Chen, Sheng Zhong, Bin He

**Affiliations:** 1 Jiangsu Key Laboratory for Environment Functional Materials, Suzhou University of Science and Technology, Suzhou 215009, China; 2 School of Materials Science and Engineering, Suzhou University of Science and Technology, Suzhou 215009, China; 3 Jiangsu Collaborative Innovation Center of Technology and Material for Water Treatment, Suzhou University of Science and Technology, Suzhou 215009, China; 4 Suzhou Institute of Environmental Science, Suzhou 215007, China; 5 CAS Key Laboratory of Green Process and Engineering, Institute of Process Engineering Chinese Academy of Sciences, Beijing 100190, China

**Keywords:** defect engineering, N_3C_ vacancies, g-C_3_N_4_, photocatalytic degradation

## Abstract

The introduction of nitrogen defects in graphitic carbon nitride (g-C_3_N_4_) has the important effect of improving its photocatalytic performance. This study employs a simple and environmentally friendly one-step pyrolysis method, successfully preparing g-C_3_N_4_ materials with adjustable N_3C_ defect concentrations through the calcination of a urea and ammonium acetate mixture. By introducing N_3C_ defects and adjusting the band structure, the conduction band of the g-C_3_N_4_ was shifted downward by 0.12 V, overcoming the traditional application limitations of N_3C_ defects and enabling an innovative transition from enhanced oxidation to enhanced reduction capabilities. This transition significantly enhanced the adsorption and activation of O_2_. Characterization results showed that the introduction of N_3C_ defects increased the specific surface area from 44.07 m^2^/g to 87.08 m^2^/g, enriching reactive sites, while narrowing the bandgap to 2.41 eV enhanced visible light absorption capacity. The g-C_3_N_4_ with N_3C_ defects showed significantly enhanced photocatalytic activity, achieving peak performance of 54.8% for tetracycline (TC), approximately 1.5 times that of the original g-C_3_N_4_, with only a 5.4% (49.4%) decrease in photocatalytic efficiency after four cycles of testing. This study demonstrates that the introduction of N_3C_ defects significantly enhances the photocatalytic performance of g-C_3_N_4_, expanding its potential applications in environmental remediation.

## 1. Introduction

With the acceleration of global industrialization and urbanization, the demand for fossil fuels has continuously increased, leading to severe environmental and energy issues [1]. Therefore, the development of green, clean, and sustainable energy sources has become an urgent global priority. Solar energy, being an abundant and clean resource, has garnered increasing attention for its potential applications in environmental remediation [2,3]. In recent years, photocatalytic technology, which can harness solar energy to degrade pollutants in water and air or convert solar energy into chemical energy, has been regarded as one of the most promising technologies for addressing energy crises and environmental pollution [4].

Graphitic carbon nitride (g-C_3_N_4_) is a non-metallic semiconductor material with a two-dimensional layered structure, unique electronic properties, and tunable optical characteristics. Due to its suitable band gap and band edge positions, it is considered one of the most promising photocatalysts [5,6,7]. This material has garnered significant attention in the field of photocatalysis due to its simple preparation method, excellent chemical and physical stability, and outstanding optical properties, enabling it to effectively absorb sunlight below 460 nm [8]. These advantages make g-C_3_N_4_ a promising semiconductor material for photocatalytic degradation of organic pollutants [9,10]. However, its photocatalytic activity is limited by a high rate of photogenerated charge carrier recombination, low light harvesting efficiency, and insufficient active sites [11,12]. To address these limitations, various modification techniques have been applied, including heterojunction construction, element doping, morphology and structural regulation, as well as defect engineering [13,14,15,16]. Among these methods, defect engineering is considered a successful approach to enhancing the photocatalytic efficiency of g-C_3_N_4_. By employing suitable strategies, defects can be introduced into the polymeric g-C_3_N_4_ which alter its electronic structure and optical properties, thereby extending its light absorption range, promoting the separation and transport of photogenerated electron–hole pairs, and enhancing its surface reactivity [17].

In recent years, defect engineering of g-C_3_N_4_ has achieved remarkable progress in enhancing its photocatalytic performance, while nitrogen [18] and carbon [19] vacancies play a critical role in improving the catalytic activity of the material. Studies on nitrogen vacancies are relatively extensive. For example, Niu et al. [20] successfully introduced nitrogen vacancies through high-temperature calcination (600 °C for 4 h), significantly altering the electronic structure of g-C_3_N_4_. Additionally, the addition of molten salts during reheating has also been used to introduce nitrogen vacancies at N_2C_ sites. For instance, Dong et al. [21] mixed melamine with molten salts (NaCl, KCl, KOH) and processed it at high temperatures to produce abundant N_2C_ vacancies. However, most studies focus on N_2C_ vacancies, with limited attention to the role of tricoordinated nitrogen (N_3C_) vacancies in photocatalysis. Theoretically, N_3C_ vacancies can provide more lone-pair electrons than N_2C_ vacancies, helping accelerate photocatalytic oxidation [22]. Liang et al. [23] created N_3C_ vacancies through self-assembly and thermal polymerization, optimizing the band structure and enhancing oxidation capacity, achieving a 97.1% degradation efficiency of ENR under visible light (NCN 0.48). Similarly, Gong et al. [24] used a hydrothermal method combined with glycerol as a reducing agent to etch the g-C_3_N_4_ surface, introducing N_3C_ vacancies, which enhanced electron–hole pair separation efficiency and increased CO_2_ reduction activity to 12 times that of GCN (4.18 μmol g^−1^ h^−1^). Despite these methods improving the photocatalytic activity of g-C_3_N_4_, they commonly involve complex pretreatments, prolonged heating, and the use of harmful reagents, making it challenging to precisely control defect concentrations [25,26]. Furthermore, products generated during photocatalytic reactions tend to adsorb onto the catalyst surface, affecting nitrogen vacancy activity and stability. Therefore, a green and straightforward method is urgently needed to synthesize highly stable g-C_3_N_4_ with N_3_C vacancies under mild conditions without harmful reagents or reducing gases.

This study synthesizes N_3C_-defect-rich g-C_3_N_4_ photocatalytic materials (denoted as ACN-X) through a simple one-step calcination method, aided by additives. Urea is mixed with ammonium acetate, and the defect concentration is controlled by adjusting the amount of ammonium acetate. During the thermal decomposition process, urea and ammonium acetate react in situ to create a reducing atmosphere, facilitating the introduction of defects into g-C_3_N_4_. Through comprehensive characterization, we examined the morphology, microstructure, light absorption capacity, and charge transfer properties of defect-rich g-C_3_N_4_ to elucidate the role of N_3C_ vacancies in photocatalysis. Additionally, the possible formation mechanism of N_3C_ vacancies during synthesis was explored, and the photocatalytic performance of the prepared g-C_3_N_4_ samples was evaluated by degrading the pollutant TC under visible light. This study achieved multiple optimizations of the material through an effective defect engineering strategy, significantly enhancing the photocatalytic activity of g-C_3_N_4_ and providing a new perspective for future environmental purification technologies.

## 2. Materials and Methods

### 2.1. Chemicals and Reagents

Ammonium acetate (C_2_H_7_NO_2_, AR), urea (H_2_NCONH_2_, AR, 99.0%), disodium ethylenediaminetetraacetate (EDTA, C_10_H_12_N_2_Na_4_O_8_·_X_H_2_O, AR, 98.0%, 0.1 M), p-benzoquinone (C_6_H_4_O_2_, 99.0%), and tert-butanol (C_4_H_10_O, AR, 99.0%) were all purchased from Aladdin Chemical Reagent Co., Ltd. (Shanghai, China). All chemicals used in this study were of analytical grade and required no further purification.

### 2.2. Photocatalytic Degradation Test

The photocatalytic performance of the material was evaluated by measuring the degradation rate of tetracycline (TC) under visible light. A 20 mg photocatalyst was added to a reactor containing 100 mL of TC solution at a concentration of 80 mg/L, with the temperature maintained at 25 °C using a circulating water system. The solution was stirred in the dark for 30 min to achieve adsorption/desorption equilibrium and then irradiated with a 300 W xenon lamp (Beijing, China) equipped with a 420 nm cutoff filter. The reaction lasted for 1 h, with samples taken every 10 min. The filtrate was analyzed using a Persee General T9 Series Double Beam UV-Vis Spectrophotometer (Beijing, China) to assess the degree of TC degradation. To test the stability of the photocatalyst, the used catalyst was washed, dried, and recovered for subsequent cycling test experiments.

### 2.3. Sample Preparation

**Scheme 1:**
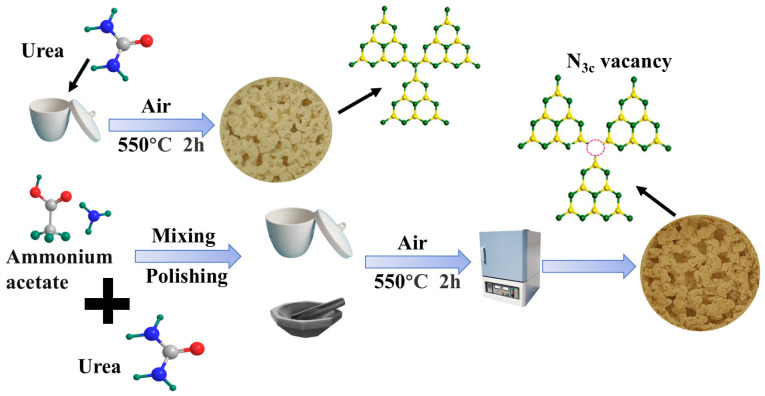
Process flow diagram for the preparation of g-C_3_N_4_ and ACN-X.

The preparation of N_3C_-vacancy g-C_3_N_4_ was processed as follows (Scheme 1): Different mass ratios of ammonium acetate (0.1–0.9 g) were mixed with urea (10 g) and synthesized using a one-step calcination method. Specifically, ammonium acetate and urea were thoroughly ground together in a mortar for 5 min, and the mixture was then placed in a crucible sealed with aluminum foil. The mixture was heated at a rate of 5 °C/min to 550 °C and maintained at that temperature for 2 h. Using this method, we successfully synthesized a series of samples labeled ACN-X, where X represents the amount of ammonium acetate added (0.1 g, 0.3 g, 0.5 g, 0.7 g, and 0.9 g). For comparative analysis, pure g-C_3_N_4_ was synthesized under the same experimental conditions using only 10 g urea.

## 3. Results and Discussion

**Figure 1:**
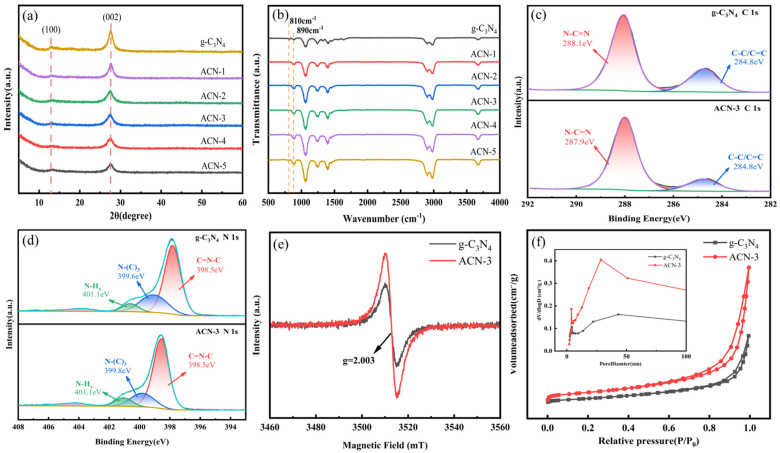
(**a**) XRD spectra of g-C_3_N_4_ and ACN-X; (**b**) FTIR spectra of g-C_3_N_4_ and ACN-X; (**c**,**d**) C 1s and N 1s XPS spectra of g-C_3_N_4_ and ACN-3. (**e**) Room temperature EPR spectra of g-C_3_N_4_ and ACN-3; (**f**) nitrogen adsorption–desorption isotherms of g-C_3_N_4_ and ACN-3 (inset shows the corresponding pore size distribution curves).

The phase structures of ACN-X and pure g-C_3_N_4_ were investigated using X-ray diffraction (XRD), as shown in Figure 1a. All samples exhibited two characteristic diffraction peaks around 13° and 27°, corresponding to the (100) and (002) crystal planes of g-C_3_N_4_, respectively. The peak at ~13° (100) is associated with the in-plane structural stacking of tri-s-triazine units, while the peak at ~27° (002) originates from the stacking of conjugated aromatic layers [27]. After the introduction of defects, no new characteristic peaks appeared or disappeared in the XRD patterns, indicating that the N_3C_ vacancy defects did not significantly alter the bulk crystalline structure of CN. However, with increased amounts of ammonium acetate, the intensity of the (100) peak at 13° slightly decreased compared to that of the original g-C_3_N_4_, suggesting a partial disruption of long-range in-plane order [28]. This implies that the introduced vacancies may slightly affect the planar structure without significantly altering the crystalline phase. Similarly, the (002) peak at 27° showed reduced intensity and slightly broadened, which may be attributed to the presence of defects within the layers. These defects might alter interlayer interactions, leading to changes in stacking density and potential increases in interlayer spacing [29]. These observations indicate that, while the overall crystal structure of g-C_3_N_4_ is retained, minor structural modifications caused by the synthesis process lead to the formation of defects that affect in-plane and interlayer structural order. This is consistent with the morphological features observed in SEM and TEM analyses.

To gain deeper insights into the chemical structure changes of the modified photocatalysts, detailed characterization was conducted using Fourier Transform Infrared Spectroscopy (FTIR). The FTIR analysis, as shown in Figure 1b, reveals that all samples exhibit the typical characteristics of graphitic carbon nitride and retain its basic structure. Specifically, the peaks at 810 cm^−1^ and 890 cm^−1^ correspond to the out-of-plane bending vibrations of the tri-s-triazine rings and the deformation vibrations of the N-H bond, respectively. The peaks in the range of 1200–1700 cm^−1^ are attributed to the stretching vibrations of N=C-N heterocycles, while the broad peaks between 3500 and 3800 cm^−1^ are primarily due to the stretching vibrations of N-H and O-H bonds, as well as adsorbed oxygen molecules [30]. With the addition of ammonium acetate, the peak intensity at 810 cm^−1^ was observed to gradually weaken and even disappear, suggesting that the structure of the heptazine rings may have been disrupted under the influence of ammonium acetate. This structural disruption could be attributed to the active radicals (NH_2_· and NH·) generated from the thermal decomposition of NH_3_ at high temperatures, which etched the N_3_C lattice sites, leading to an increase in N_3C_ vacancies and partial degradation of the heptazine rings [31]. These changes indicate the successful introduction of N_3C_ vacancies, and the greater the amount of ammonium acetate added, the more nitrogen vacancies were formed.

X-ray photoelectron spectroscopy (XPS) was performed to analyze the surface elemental composition, chemical states, and defect types after the addition of ammonium acetate. As shown in Figure S1, the presence of C and N elements is identified in both g-C_3_N_4_ and ACN-3, while the O element is likely due to surface adsorption occurring during the calcination process. As shown in Figure 1c, the high-resolution XPS spectra of C 1s display two main peaks at 284.8 eV and 288.1 eV, corresponding to graphitic carbon at the edges of triazine rings (C=C/C-C) and sp^2^ hybridized carbon (N–C=N), respectively. Notably, the peak at 288.1 eV shifts to a lower binding energy (287.9 eV), indicating that sp^2^ hybridized carbon has gained extra electrons from the missing nitrogen atoms, which partially confirms the presence of nitrogen vacancies [32]. In the high-resolution XPS spectra of N 1s (Figure 1d), there are four characteristic deconvolution peaks located at 398.5 eV (N_2C_), 399.6 eV (N_3C_), 401.1 eV (N-H_X_), and 404.1 eV (charge effects in heterocycles). For ACN-3, all peaks show no significant shift. Moreover, based on the XPS fitting analysis, the peak area of N_3C_ decreases in ACN-3 after the addition of ammonium acetate, and the N_3C_/N_2C_ peak area ratio decreases from 0.41 to 0.35. It is noteworthy that the N_3C_/N-H_X_ ratio in both pure g-C_3_N_4_ and ACN-3 remains almost unchanged, suggesting that vacancies preferentially form at N_3C_ sites [33].

To investigate in detail how the defect concentration of N_3C_ vacancies changes with the increase in the amount of ammonium acetate, we conducted high-resolution XPS analysis of the C1s and N1s spectra of different samples (Figure S2) and examined the variations in the content of various chemical bonds in the ACN-X series samples (see Table 1 and Table 2). The results show that with the increased addition of ammonium acetate, the content of N_3C_ in the samples gradually decreased, indicating that the nitrogen atoms at the N_3C_ sites are more prone to being replaced or removed, leading to an increase in nitrogen vacancies. Meanwhile, the relative increase in the content of N_2C_ suggests that after the nitrogen at N_3C_ sites is removed, the surrounding structure may rearrange, causing some nitrogen atoms to transition to N_2C_ coordination forms. This structural change particularly affects the bonding state of nitrogen atoms within the conjugated triazine framework, resulting in alterations in the electron distribution of the N=C=N structure. The enhancement of the infrared absorption peak corresponding to the N=C=N stretching vibrations (1700–1200 cm^−1^) in the FTIR spectra further confirms that significant changes in the surface and chemical structure of the material occurred due to the introduction of defects. Additionally, the gradual decrease in the N_3C_/N_2C_ peak area ratio, from 0.41 in g-C_3_N_4_ to 0.32 in ACN-5, further confirms that the number of N_3C_ vacancies increases with the increased addition of ammonium acetate [34]. Therefore, it can be concluded that the defect concentration increases as the amount of ammonium acetate added is increased.

The C1s spectral data also reveal that the content of graphitic carbon (C=C/C-C) decreases with the increased addition of ammonium acetate. This may be due to the disruption of the original arrangement of carbon atoms connected to N_3C_ during the formation of nitrogen vacancies, leading to damage in the graphitized regions of the material. The reduction in the degree of graphitization implies a decline in the orderliness of the interlayer structure, resulting in a decrease in the intensity of the (002) peak and an increase in peak width in the XRD analysis. This indicates a more disordered crystalline structure and a higher defect rate, consistent with the crystalline changes observed in the XRD results.

During the reaction between urea and ammonium acetate to produce carbon nitride materials, although nitrogen vacancies were formed, the mechanism differs from the traditional nitrogen vacancy formation (i.e., reduction in nitrogen atoms). The C/N ratio shows a downward trend with increasing amounts of ammonium acetate (Table 3). This may be because, under high-temperature conditions, the reaction between ammonium acetate and urea not only promotes the formation of defects at N_3C_ sites but also leads to the adsorption of some nitrogen-containing groups (such as NH_X_) on the material surface in a closed environment. Additionally, the presence of free amino groups may facilitate the formation of N-H_X_ bonds, leading to an increase in nitrogen content in the final product. This also accounts for the slight increase in N-H_X_ content. The NHx groups adsorbed on the material’s surface influence the surface charge distribution, enhance hydrophilicity, and facilitate the desorption of reaction products (such as intermediates or final products), thereby reducing their accumulation on the surface. This helps maintain the accessibility of active sites and enhances the stability of photocatalysis. In summary, the analysis indicates that by adjusting the reaction conditions (particularly the amount of ammonium acetate added), it is possible to effectively regulate the defect concentration in the material, which also triggers other surface chemical and structural changes, thereby affecting the overall photocatalytic performance of the material.

The presence of nitrogen vacancies was further confirmed by electron paramagnetic resonance (EPR) spectroscopy. As shown in Figure 1e, the EPR spectra of pure g-C_3_N_4_ and ACN-3 exhibit similar single Lorentzian lines, with a g-value of 2.003, indicating the presence of unpaired electrons in sp^2^ hybridized carbon atoms within the π-conjugated aromatic rings [35]. Compared to pure g-C_3_N_4_, the EPR signal of the material with introduced defects is significantly enhanced, which is attributed to the effective increase in unpaired electron concentration due to the introduction of N_3C_ vacancies, thereby providing more free electrons to the sp^2^ carbon atoms [36]. This increase in unpaired electrons facilitates the generation and separation of charge carriers in the material, promoting the efficient transfer of photogenerated electrons. Based on the combined results of EPR and XPS spectra, the introduction of N_3C_ vacancies has been confirmed to successfully modulate the electronic structure of carbon nitride, enhancing the concentration of unpaired electrons, which is expected to improve its activity and efficiency in photocatalytic reactions.

Based on the analysis results, a mechanism for the formation of nitrogen vacancies in ACN-3 is proposed. During the heating phase between 110 and 210 °C (Stage 1), ammonium acetate partially decomposes, releasing NH_3_ and CO_2_. Simultaneously, partial decomposition products of acetic acid and urea interact, leading to an acylation reaction that forms amide compounds. As the temperature continues to rise, the amide compounds gradually decompose, releasing a large volume of gaseous products (CO_2_ and NH_3_) and creating a self-generated reducing atmosphere. Under high-temperature conditions, the generated NH_3_ decomposes into reactive radicals (such as NH_2_· and NH·), which can effectively etch the N_3C_ lattice sites in g-C_3_N_4_, participating in the reaction within the carbon nitride material and promoting the formation of nitrogen vacancies. Furthermore, the continuous release of gaseous products introduces a porous structure into the material, thereby increasing the specific surface area and further enhancing the material’s reactivity and photocatalytic performance.

The N_3C_ defect-engineered g-C_3_N_4_ photocatalysts were synthesized via a one-step copolymerization method by uniformly mixing ammonium acetate and urea. During the thermal polycondensation process, ammonium acetate reacts with urea through an acylation reaction, accompanied by the release of gases (CO_2_ and NH_3_) and volume contraction, which induces the transformation of the morphology from irregular bulk structures to porous amorphous sheet-like structures [7,37,38,39]. To verify the effect of defect engineering on the morphological structure, the synthesized catalysts were characterized by scanning electron microscopy (SEM) and transmission electron microscopy (TEM) to examine their morphology and microstructure.

**Figure 2:**
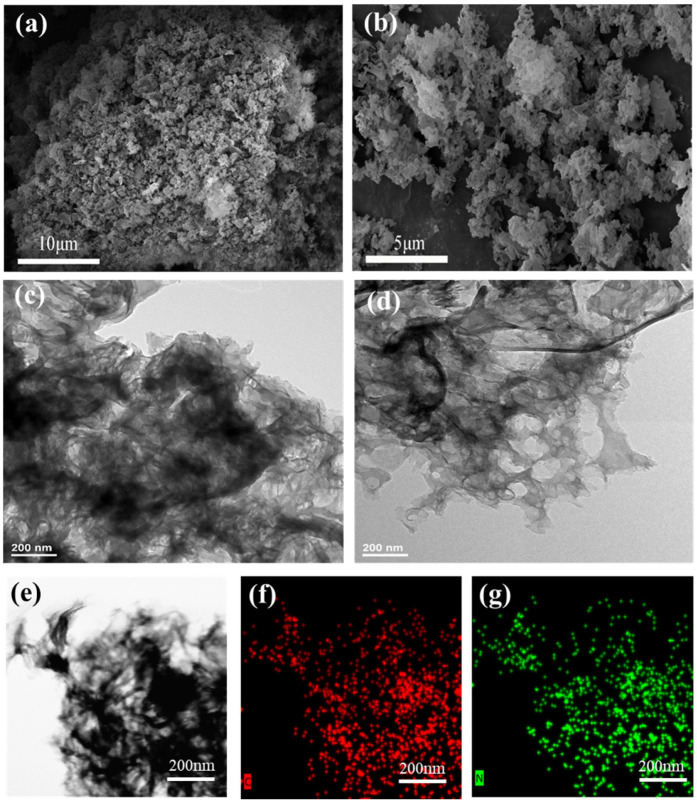
(**a**,**b**) SEM images of g-C_3_N_4_ and ACN-3; (**c**,**d**) TEM images of g-C_3_N_4_ and ACN-3; (**e**) HAADF STEM image; and (**f**) EDX elemental distribution maps of carbon (C) and (**g**) nitrogen (N) in ACN-3.

From the SEM images of g-C_3_N_4_ and ACN-3 (Figure 2a,b), it can be observed that under the influence of ammonium acetate, g-C_3_N_4_ transforms from a bulk structure into an amorphous, layered, porous structure. Compared to pure g-C_3_N_4_, ACN-3 exhibits a looser morphology and enhanced porosity. The TEM images of g-C_3_N_4_ and ACN-3 (Figure 2c,d) further demonstrate ACN-3’s distinctive porous structure, featuring stacked, sheet-like morphology with numerous in-plane pores. In addition, the HAADF-STEM and EDX elemental distribution maps in Figure 2a,b indicate that in ACN-3, the elements C and N are uniformly distributed within the selected area. To further investigate the surface characteristics of g-C_3_N_4_ and ACN-3, BET analysis was performed. The N_2_ adsorption–desorption isotherms and pore size distribution curves (Figure 1f) reveal that both materials exhibit typical type IV isotherms with H3 hysteresis loops, indicating the formation of mesoporous and macroporous structures, consistent with the SEM and TEM results. Compared to g-C_3_N_4_, the modified ACN-3 shows a higher specific surface area and pore volume (Table 4), along with a diverse pore structure ranging from micropores to macropores. The pore sizes of both catalysts are primarily distributed in the 2–40 nm range, suggesting the formation of numerous mesopores within the material. The introduction of defects did not lead to the collapse of the mesoporous structure but endowed ACN-3 with a more loosely packed morphology and rich porous characteristics. The increase in specific surface area provides more active sites on the catalyst surface [40], facilitating the adsorption of reactants and the desorption of products. These active sites can disperse the reaction centers, preventing the excessive accumulation of intermediates in localized regions and thereby reducing the shielding of active nitrogen vacancies. Simultaneously, this also promotes the diffusion and transport of reactants and products on the catalyst surface, accelerating the reaction process and enhancing the effective adsorption of pollutants and the photocatalytic performance [41].

The introduction of defects did not lead to the collapse of the mesoporous structure. Instead, it endowed ACN-3 with a looser morphology and abundant porosity, providing more active sites, which aids in the efficient adsorption of pollutants and enhances photocatalytic performance.

**Figure 3:**
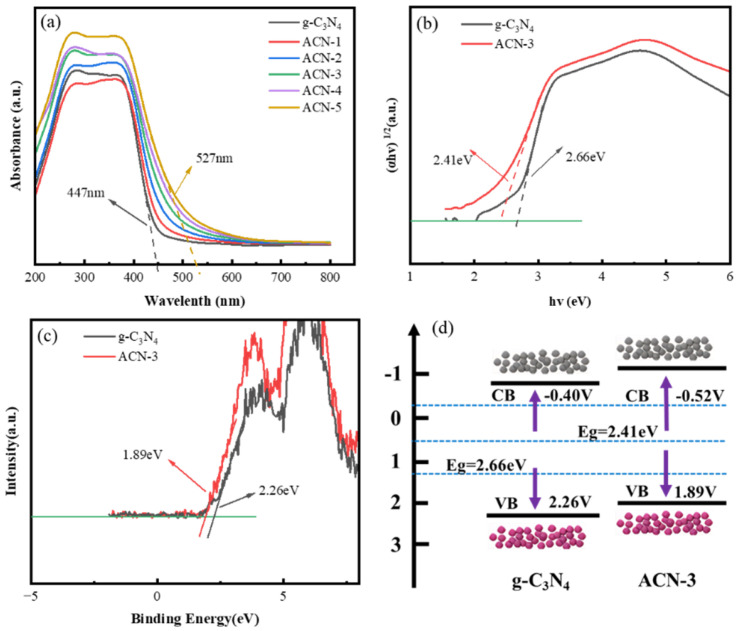
(**a**) UV–visible absorption spectra of g-C_3_N_4_ and ACN-X; (**b**) transformation of the Kubelka–Munk function versus photon energy for g-C_3_N_4_ and ACN-3; (**c**) XPS valence band spectra of g-C_3_N_4_ and ACN-3; and (**d**) the corresponding band structure diagrams.

The influence of N_3C_ vacancies on the electronic band structure and optical properties of g-C_3_N_4_ was analyzed through UV-Vis diffuse reflectance spectroscopy (UV-Vis DRS). As shown in Figure 3a, pure g-C_3_N_4_ exhibits typical semiconductor absorption characteristics in the UV and part of the visible light region, with an absorption edge around 447 nm. In contrast, ACN-3 shows a significant redshift in the absorption edge (484 nm), and with the increasing amount of ammonium acetate, all samples exhibit further redshift (527 nm), indicating stronger visible light absorption. This phenomenon is likely due to the introduction of N_3_C vacancies, which creates intermediate band gaps in g-C_3_N_4_, enhancing both light absorption and charge transfer efficiency. In addition, the decomposition products of ammonium acetate disrupt the polymerization process of carbon nitride, introducing defects that lead to the distortion of its 2D planar structure, activating the n → π* electronic transition, and significantly broadening the light absorption range, particularly in the visible light region, thereby enhancing photocatalytic performance [42,43,44]. Figure 3b shows the relationship between the Kubelka–Munk function and photon energy for g-C_3_N_4_ and ACN-3. Based on the Kubelka–Munk theory, the band gap of ACN-3 is estimated to be 2.41 eV, lower than that of pure g-C_3_N_4_ 2.66 eV, indicating that ACN-3 has a broader light absorption range. With the increased addition of ammonium acetate, the rising concentration of N_3C_ vacancies further narrows the band gap, enabling wide-range tuning of the energy band (Figure S3). This provides advantageous conditions for improving the efficiency of the photocatalyst in the visible light region.

Furthermore, the valence band position of ACN-3 (Figure 3c) and its band structure (Figure 3d) were analyzed through XPS valence band spectroscopy. Compared with pure g-C_3_N_4_, the introduction of N_3_C vacancies results in a more negative conduction band potential in ACN-3, indicating that its photogenerated electrons possess a stronger reducing ability, allowing for more efficient generation of ·O_2_^−^ radicals, which can effectively initiate photocatalytic reduction reactions [28]. Additionally, the narrower band gap of ACN-3 more effectively reduces the recombination of photogenerated electrons and holes, achieving enhanced spatial charge separation, which extends the lifetimes of these charge carriers. This improvement in photocatalytic activity provides a strong structural basis for increased photocatalytic efficiency [23].

**Figure 4:**
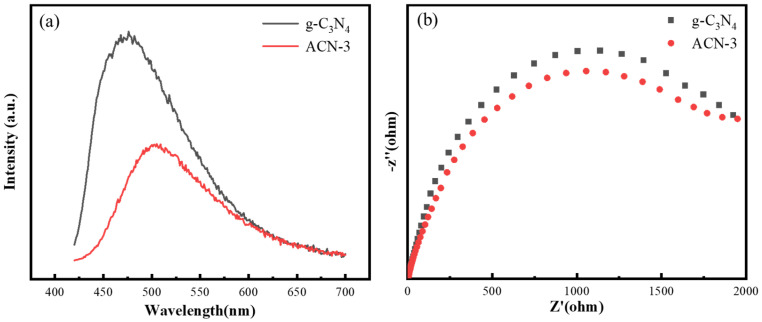
(**a**) Nyquist plots from EIS measurements, and (**b**) PL spectra of g-C_3_N_4_ and ACN-3.

To further investigate the effect of N_3C_ vacancies on the efficiency of photogenerated charge separation and optical properties, we conducted electrochemical impedance spectroscopy (EIS) measurements on g-C_3_N_4_ and ACN-3. The EIS results (Figure 4a) show that, compared to pure g-C_3_N_4_, the arc radius in the impedance response of ACN-3 is significantly reduced, indicating lower surface resistance and faster interfacial charge transfer. These characteristics suggest that N_3C_ vacancies greatly facilitate the transport of photogenerated electrons from the interior of the material to the surface, accelerating the migration of interfacial charges. Additionally, photoluminescence (PL) spectroscopy was used to further examine the behavior of photogenerated carriers [45]. At an excitation wavelength of 365 nm, the PL intensity of ACN-3 is much lower than that of pristine g-C_3_N_4_ (Figure 4b). This weaker PL signal indicates a significant reduction in the recombination rate of photogenerated charges, primarily due to the presence of nitrogen vacancies. The nitrogen vacancies help trap photogenerated holes, reducing the spatial overlap between photogenerated carriers and enhancing their separation efficiency [41,46]. Therefore, it can be inferred that the presence of N_3C_ vacancies effectively suppresses the recombination of photogenerated carriers, thereby enhancing their transport and photocatalytic utilization efficiency. Through these measurements, we have confirmed the crucial role of N_3C_ vacancies in improving the photoelectric performance of the material.

**Figure 5:**
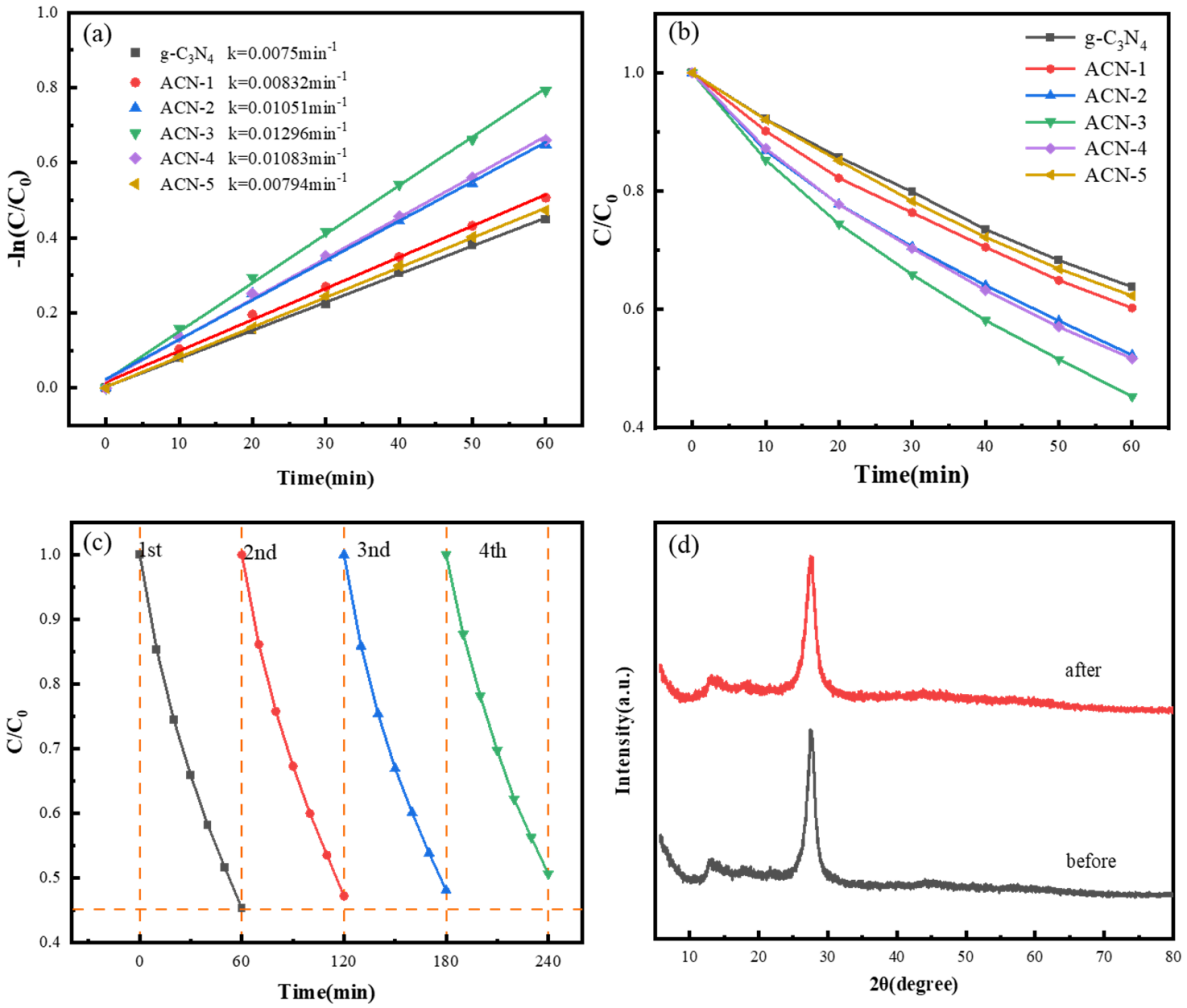
(**a**) First-order kinetic curves for the degradation of TC; (**b**) photocatalytic degradation of TC by samples with varying ratios; (**c**) four-cycle photocatalytic degradation of TC using ACN-3; and (**d**) XRD patterns of ACN-3 before and after the photocatalytic experiment.

The photocatalytic performance of the prepared catalysts was evaluated using TC as the target pollutant. As shown in Figure 5a, the results indicated a significant improvement in the photocatalytic degradation efficiency of ACN-X samples with different defect concentrations compared to pure g-C_3_N_4_. The degradation rate of pure g-C_3_N_4_ was only 36.2%, while the ACN-X samples showed varying degrees of enhanced efficiency based on the defect concentration. A defect concentration that is too low cannot effectively inhibit the recombination of photogenerated electron–hole pairs, which is essential for improving the separation and transfer efficiency of charge carriers. Conversely, an excessively high defect concentration may lead to electron accumulation, forming recombination centers for charge carriers, which suppresses photocatalytic efficiency [17]. Therefore, precise control over the defect concentration is crucial. When the defect concentration was optimized, the degradation efficiency of ACN-3 reached 54.8%, which is 1.5 times that of pure g-C_3_N_4_. This significant improvement is primarily attributed to the introduction of N_3C_ vacancies through defect engineering. These vacancies effectively reduced the recombination of photogenerated electron–hole pairs, enhancing the separation and transport efficiency of charge carriers. Moreover, the introduction of defects increased the specific surface area of the material and formed a rich porous structure, providing more active sites for the photocatalytic reaction, further promoting pollutant adsorption and improving the efficiency of photocatalytic degradation.

Figure 5b shows the first-order reaction rate constants of the samples with different defect concentrations. The reaction rate constant of pure g-C_3_N_4_ is 0.0075 min^−1^, while the rate constant of ACN-3, with the optimal defect concentration, increases to 0.0129 min^−1^, approximately 1.72 times that of unmodified g-C_3_N_4_. This indicates that the rational introduction of N_3C_ vacancy defects effectively altered the electron distribution on the surface of g-C_3_N_4_, enhancing the transport efficiency of photogenerated electrons from the interior to the surface of the material, thereby significantly accelerating the photocatalytic reaction rate. Figure 5c presents the cyclic degradation performance of ACN-3 at the optimal defect concentration. According to the calculations, the photocatalytic degradation efficiency of ACN-3 in the first cycle was 54.8%. After four consecutive cycles, the efficiency remained at around 49.4%. This indicates that the material maintained high photocatalytic performance during repeated use, demonstrating excellent cyclic stability. Additionally, a comparison of the XRD patterns of ACN-3 before and after the reaction (Figure 5d) shows no significant changes in its crystal structure. The stability of the material can be attributed to its robust structure and the introduction of N_3C_ vacancies through defect engineering, which effectively prevented the loss of active sites. This ensured that the photocatalytic efficiency remained high even after multiple uses, enhancing its feasibility for practical applications.

**Figure 6:**
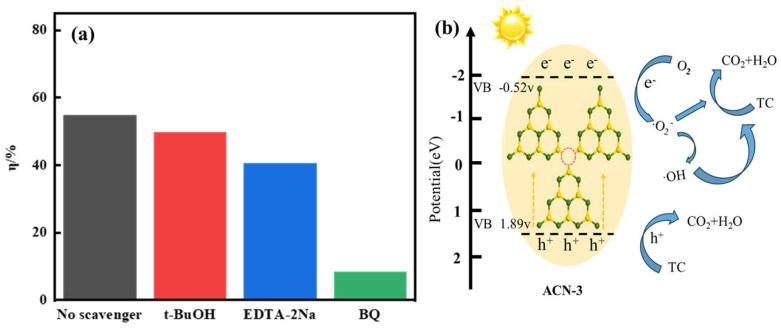
(**a**) Radical scavenging experiments for TC degradation by ACN-3 under visible light; (**b**) proposed reaction mechanism for TC degradation by ACN-3 under visible light irradiation.

We conducted a radical scavenging experiment by adding quenching agents to investigate the photocatalytic mechanism of ACN-3. In the experiment, t-BuOH was used as a scavenger for ·OH, BQ was used to capture ·O_2_^−^, and EDTA-2Na was used to capture h^+^ [47]. As shown in Figure 6a, the photocatalytic degradation of TC was significantly inhibited when ·O_2_^−^ scavenger BQ and h^+^ scavenger EDTA-2Na were added, reducing the degradation efficiency to 8.3% and 40.5%, respectively. In contrast, t-BuOH had a negligible effect on TC degradation. This indicates that hydroxyl radicals (·OH) are not the main active species in the photocatalytic reaction and play a relatively limited role. Instead, superoxide radicals (·O_2_^−^) act as the key active species, dominating the reaction, while the influence of h^+^ is secondary.

Based on the above findings, we propose a photocatalytic mechanism for g-C_3_N_4_ containing N_3C_ vacancies, as illustrated in Figure 6b, which effectively explains its enhanced activity during the photocatalytic process. The introduction of N_3C_ vacancies not only decreases the electron density of g-C_3_N_4_ but also enhances intersystem transition capability, facilitating the generation of photogenerated electrons and holes. Additionally, N_3C_ vacancies serve as trap states for photogenerated electrons, effectively promoting the separation and transfer of electrons and holes, significantly reducing their recombination rate. Under visible light irradiation, photoexcitation of ACN-3 generates electron–hole pairs with a more negative conduction band potential, making its photogenerated electrons more prone to reacting with molecular oxygen to generate ·O_2_^−^ radicals, which play a crucial role in the reaction. Concurrently, photogenerated holes (h^+^) also participate in the reaction by directly interacting with TC, catalyzing its oxidative decomposition into carbon dioxide (CO_2_) and water (H_2_O). Table 5 provides a summary comparison of recent studies with our work.

## 4. Conclusions

This study successfully synthesized g-C_3_N_4_ (ACN-X) with tricoordinated nitrogen vacancies (N_3C_) using a simple one-step calcination process assisted by additives. This method requires no harmful reducing agents, simplifying the preparation and defect generation process while enhancing the reproducibility and stability of the material. The defect engineering significantly optimized the electronic structure of the material, reducing the bandgap from 2.66 to 2.41 eV, thereby enhancing light absorption and energy capture efficiency, achieving effective separation of photogenerated electrons and holes, and reducing recombination rates. By precisely tuning the band structure, this study innovatively transformed the N_3C_-defect g-C_3_N_4_ material from enhanced oxidation capability to enhanced reduction capability, significantly improving its photocatalytic reduction performance. Under low dosage (20 mg), high concentration (80 mg/L, 100 mL), and short duration (1 h), ACN-3 achieved a TC degradation rate of 54.8%, with only a 5.4% decline in degradation efficiency after four cycles. This study provides new insights into the potential of carbon nitride materials in reductive photocatalytic applications.

## Figures and Tables

**Table 1:** Relative proportions of different chemical bonds in the N1s spectra of g-C_3_N_4_ and ACN-X samples.

Sample	N_2C_ (at %)	N_3C_ (at %)	N-H_X_ (at %)	N_3C_/N_2C_ (at %)
g-C_3_N_4_	39.30	16.20	4.44	0.41
ACN-1	40.48	14.58	4.49	0.36
ACN-3	41.16	14.50	4.51	0.35
ACN-5	43.04	14.05	4.76	0.32

**Table 2:** Relative proportions of different chemical bonds in the C1s spectra of g-C_3_N_4_ and ACN-X samples.

Sample	C=C/C-C (at %)	N–C=N (at %)
g-C_3_N_4_	11.40	24.63
ACN-1	12.48	24.77
ACN-3	10.60	23.75
ACN-5	9.79	24.54

**Table 3:** Elemental composition of g-C_3_N_4_ and ACN-X samples.

Sample	C%	N%	C/N
g-C_3_N_4_	50.41	49.59	1.01
ACN-1	49.72	50.28	0.98
ACN-3	48.75	51.28	0.95
ACN-5	48	52	0.92

**Table 4:** Specific surface area and pore structure parameters of g-C_3_N_4_ and ACN-3.

Name	S_BET_ (m^2^/g)	Pore Volume (cm^3^/g)	Average Pore Size (nm)
g-C_3_N_4_	44.47	0.1898	17.0696
ACN-3	87.08	0.3725	17.1123

**Table 5:** Photocatalytic activity for the TC degradation using carbon nitride in previous studies.

Samples	Dosage (mg)	Pollutants	Reaction Time	Reaction Conditions	Light Source	Removal Rate	Ref.
NCN 0.48	40	40 mL ENR (20 mg/L)	240 min	H_2_O	Xenon lamp (300 W, λ > 420 nm)	97.1%	[23]
Ht CN	50	50 mL TC (20 mg/L)	80 min	H_2_O	Xenon lamp (300 W, λ > 420 nm)	67%	[46]
CN1.5	50	200 mL TC (30 mg/L)	75 min	H_2_O	Xenon lamp (300 W, λ > 420 nm)	87.9%	[48]
CNx-0.5	20	50 mL TC (10 mg/L)	60 min	PS (2 mM, PH: 7)	Xenon lamp (300 W, λ > 420 nm)	68%	[49]
M-CN	100	50 mL TC (15 mg/L)	120 min	H_2_O	Xenon lamp (300 W, λ > 420 nm)	82%	[50]
P_0.3_S_0.2_-CN	30 mg	50 mL (20 mg/L)	100 min	1.6 mg FeSO_4_·_7_H_2_O	Xenon lamp (300 W, λ > 420 nm)	87.3%	[51]
ACN-5	100 mg	100 mL (10 mg/L)	60 min	H_2_O	Xenon lamp (250 W, λ > 420 nm)	88%	[52]
g-C_3_N_4–0.5_/g-c_3_N_4_-2	30	50 mL TC (20 mg/L)	120 min	H_2_O	Xenon lamp (300 W, λ > 420 nm)	84.2%	[53]
Br/K/cyano-CN	10	50 mL (10 mg/L)	30 min	Fe^2+^ (4 mg/L)/H_2_O_2_	Xenon lamp (300 W, λ > 420 nm)	97.3%	[54]
CN0.75	20	50 mL (20 mg/L)	60 min	H_2_O	Xenon lamp (300 W, λ > 420 nm)	93%	[55]
20TaON/KPHI	20	30 mL (10 mg/L)	30 min	H_2_O	Light emitting diode (LED, M455F3)	97%	[56]
ACN-3	20	100 mL TC (80 mg/L)	60 min	H_2_O	Xenon lamp (300 W, λ > 420 nm)	54.8%	This work

## Data Availability

The data that support the findings of this study are available in the Supplementary Information and from the corresponding author upon reasonable request.

## Supplementary Materials

The following supporting information can be downloaded at: https://www.mdpi.com/article/10.3390/nano15060466/s1, Figure S1: XPS spectra of g-C_3_N_4_ and ACN-3; Figure S2: XPS spectra of C 1s and N 1s for ACN-1 and ACN-5; Figure S3: The Kubelka–Munk function versus photon energy conversion plot for g-C_3_N_4_ and ACN-X.
