# Supplementary material for: N3C-Defect-Tuned g-C3N4 Photocatalysts: Structural Optimization and Enhanced Tetracycline Degradation Performance
Source: Nanomaterials (Basel). 2025 Mar 19;15(6):466. doi: 10.3390/nano15060466 (PMC11946266; doi:10.3390/nano15060466)
Supplement: Supplementary file 1 [file nanomaterials-15-00466-s001.zip › nanomaterials-3519067-supplementary.pdf]

Supporting Information

N_{3C} Defect-Tuned g-C_3N_4 Photocatalysts: Structural Optimization and Enhanced Tetracycline Degradati on Performance

1. Measurements and Characterizations

The crystal structure of the powders was analyzed using X-ray diffraction (XRD) with a Rigaku D/max-2400 X-ray diffractometer utilizing CuKα radiation (40 kV, 20 mA; λ = 1.5406 Å) over a scanning range of 10-80°. The morphology of the products was examined by scanning electron microscopy (SEM, Hitachi S-3500 N) and transmission electron microscopy (TEM, JEOL JEM2011 F). UV–vis absorption spectra of the samples were obtained using a Hitachi UV-3010 spectrophotometer with $BaSO_4$ as the reference material. Fourier transform infrared (FT-IR) spectra were recorded on a Bruker VERTEX-70 spectrometer. X-ray photoelectron spectroscopy (XPS) measurements were performed on a PHI Quantera SXM equipped with a monochromatized Al Kα X-ray source (pass energy 55 eV). The specific surface area and pore distribution of the samples were evaluated by N_2 adsorption/desorption using a Micromeritics ASAP2010 V5.02H. The steady and transient photoluminescence (PL) curves of the products were obtained using an Edinburgh Instruments FLS980 fluorescence lifetime spectrophotometer. Electron paramagnetic resonance (EPR) signals of the photocatalysts were acquired using a JEOL JES FA200 spectrometer at room temperature. Photoelectrochemical Measurements: Electrochemical impedance spectroscopy (EIS) was conducted using a three-electrode system on a Shanghai Chenhua CHI660D electrochemical workstation.

2. Photocatalytic Degradation Test

The photocatalytic properties of the materials were evaluated based on the degradation rate of tetracycline (TC) under visible light. The photocatalytic experiment was conducted using a circulating water system to maintain a constant reaction temperature of 25°C. The photocatalyst (20 mg) was placed in a reactor containing 100 mL of TC solution (80 mg/L). After connecting the setup, the system was illuminated by a 300W xenon lamp equipped with

a 420 nm cut-off filter. Prior to illumination, the solution was stirred in the dark for 30 minutes to ensure adsorption/desorption equilibrium. The light source was then turned on to initiate the photocatalytic degradation reaction for 1 hour, with sampling every 10 minutes. The filtrate was analyzed using a Shimadzu UV-2450 spectrophotometer, and the concentration of the solvent post-photocatalysis was determined by recording the change in absorbance at the maximum wavelength.

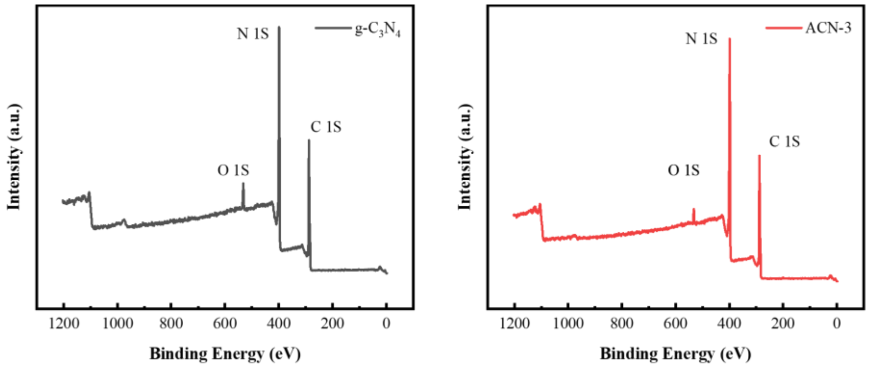

Fig. S1. XPS spectra of g-C3N4 and ACN-3.

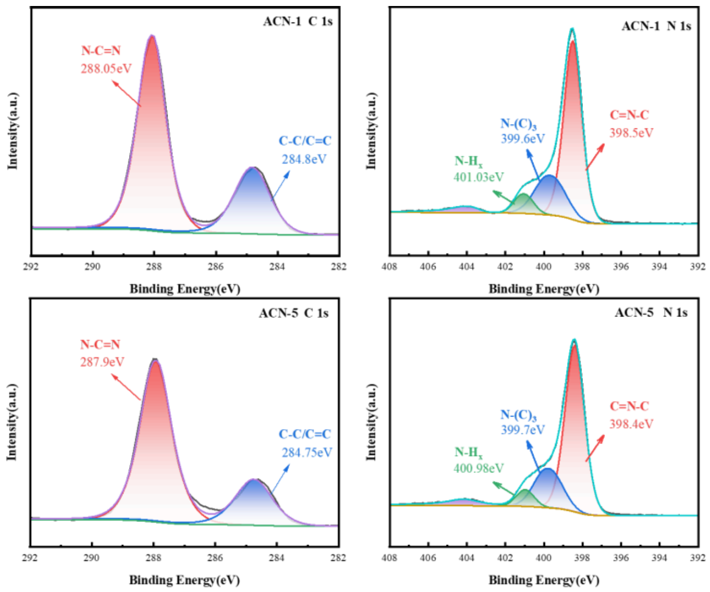

Fig. S2. XPS spectra of C 1s and N 1s for ACN-1 and ACN-5.

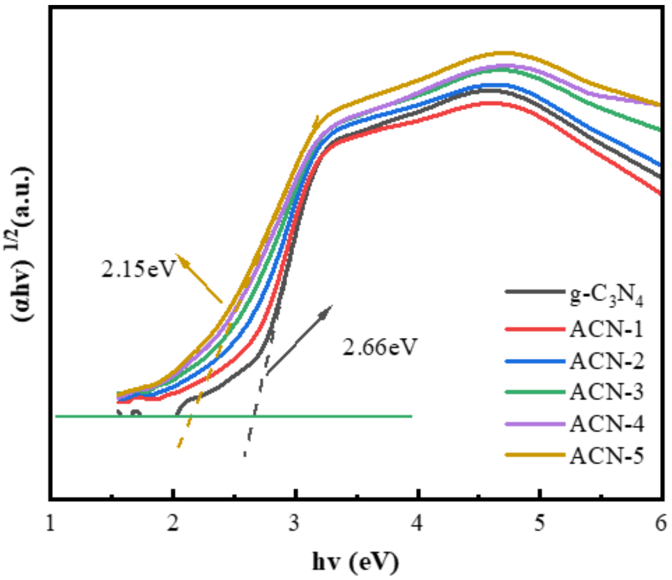

Fig. S3. shows the Kubelka-Munk function versus photon energy conversion plot for g-C_3N_4 and ACN-X.
